# Performance Properties of Epoxy Resin Modified with Few-Layer Graphene Obtained by the Method of Self-Propagating High-Temperature Synthesis

**DOI:** 10.3390/polym17060812

**Published:** 2025-03-20

**Authors:** Nikita Podlozhnyuk, Aleksei Vozniakovskii, Sergey Kidalov, Alexander Voznyakovskii

**Affiliations:** 1 Ioffe Institute RAS, Politekhnicheskaya St., 26, St. Petersburg 194021, Russia; podloznuknikita@gmail.com (N.P.); alexey_inform@mail.ru (A.V.); 2 Scientific Research Institute of Synthetic Rubber Named After Academician S. V. Lebedev, Gapsalskaya St., 1, St. Petersburg 198035, Russia; voznap@mail.ru

**Keywords:** graphene, few-layer graphene, epoxy resin, strength, wear resistance, thermal conductivity, glass transition

## Abstract

This study presents the results of an investigation into the influence of few-layer graphene, produced by self-propagating high-temperature synthesis from various types of biopolymers (glucose, cellulose, and lignin), on the mechanical, thermophysical, and tribological properties of epoxy resin. It was found that the addition of few-layer graphene at concentrations of up to 1 wt.% leads to an increase in compressive strength by up to 40%, flexural strength by up to 15%, and thermal conductivity by up to 40% compared to the original resin. A fivefold increase in the wear resistance of the composites was also observed compared to pure epoxy resin, due to a reduction in the friction coefficient.

## 1. Introduction

Epoxy resin is one of the most widely used polymer materials and has many applications. Epoxy resin is a polymer used as protective coatings, construction materials, adhesives, etc. [1]. At the same time, developing epoxy resins with new performance properties that could meet the growing modern requirements becomes an increasingly difficult task due to the limited scope of possible combinations of various components [2]. Researchers are increasingly resorting to creating composite materials based on epoxy resins with multiple fillers to improve the performance characteristics of already used brands of epoxy resins. Graphene nanostructures (GNS) are one of the most popular fillers for creating composites based on epoxy resins [3].

Interest in GNS is due to their characteristics: the thermal conductivity of defect-free single-layer graphene is up to 5000 W/(m × K) [4]; its Young’s modulus and tensile strength are up to 1 TPa and up to 130 GPa, respectively [5]. Depending on the structure of GNS (lateral size, number of layers, defects, etc.), they inherit the record-breaking characteristics of graphene to one degree or another.

Based on the principle of creating composite materials, the use of GNS as a modifying additive even in small quantities (units of weight %) should lead to a significant increase in the properties of the epoxy composite [6].

Studies have shown that the final properties of epoxy composites are influenced by the characteristics of the GNS, such as their chemical composition [7,8], number of layers [9], composition of surface groups [10], lateral particle sizes [11], and defectiveness [12]. It is also necessary to take into account the influence of the methods used for distributing the GNS in the volume of the epoxy matrix (mechanical mixing, ultrasonic treatment, etc.) [13].

Although the actual efficiency of GNS in creating epoxy composites turned out to be significantly lower than theoretical expectations [14,15,16], the use of GNS allows to significantly improve the properties of epoxy composites. However, GNS are still not used in the renal industry, including for economic reasons [17]. The lack of implementation of scientific results in the industry is due to the imperfection of methods for synthesizing GNS both according to the “bottom-up” approach and the “top-down” approach [18,19], which do not allow synthesizing large volumes of high-quality material with a low cost.

One of the key tasks of researchers is to search for new methods for synthesizing GNS that will solve these problems. One of the latest promising approaches to synthesizing GNS is self-propagating high-temperature synthesis (SHS). The essence of the method is to obtain new substances during the movement of a wave of a strong exothermic reaction, in which heat release is localized in a narrow layer and is transmitted from layer to layer by heat transfer [20]. Initially, this method was used to obtain various inorganic compounds, such as carbides of refractory metals [21]. However, scientists also found that it is possible to synthesize GNS under SHS conditions.

In [22], the authors used a mixture of magnesium powder and polyvinyl alcohol/glucose/calcium carbonate/magnesium carbonate powder to synthesize GNS, which was initiated using a red-hot filament in a carbon dioxide atmosphere. The authors of this work have shown that GNS can be obtained from calcium and magnesium carbonate, glucose, and polyvinyl alcohol.

In our previous work [23], demonstrated a new method for synthesizing few-layer graphene (material consisting of 3–10 graphene layers [24]) under SHS conditions from cyclic biopolymers. This method makes obtaining large volumes of few-layer graphene possible, which do not contain Stone–Wales (SW) defects in their structures [25]. Due to this feature, few-layer graphene obtained by the SHS method has demonstrated greater efficiency as a modifying additive in creating products from photopolymer resins by Digital Light Processing (DLP) 3D-printing compared to GNS containing SW defects [26].

This work aimed to study the effectiveness of using few-layer graphene synthesized under SHS conditions from glucose, cellulose, and lignin as a modifying additive in creating polymer composites based on epoxy resin.

## 2. Materials and Methods

FLG synthesized under SHS conditions from glucose, cellulose (saccharides), and lignin (polyphenol) was used as a modifying additive. Detailed information on the equipment used, reagent ratios, reaction temperatures, etc., is presented in [23]. Graphene oxide (GO) synthesized by a modified Hammers method [27], which was treated with hydrazine to obtain reduced graphene oxide (rGO), was also taken as a modifying additive. Epoxy resin KER-828 (Kumho P&B Chemicals, Seoul, Republic of Korea) with a mass fraction of epoxy groups of 0.53 mol/g and hardener triethylenetetramine (TETA) were used to obtain the composites, the ratio of epoxy resin to hardener was 10/1.

**Figure 1:**
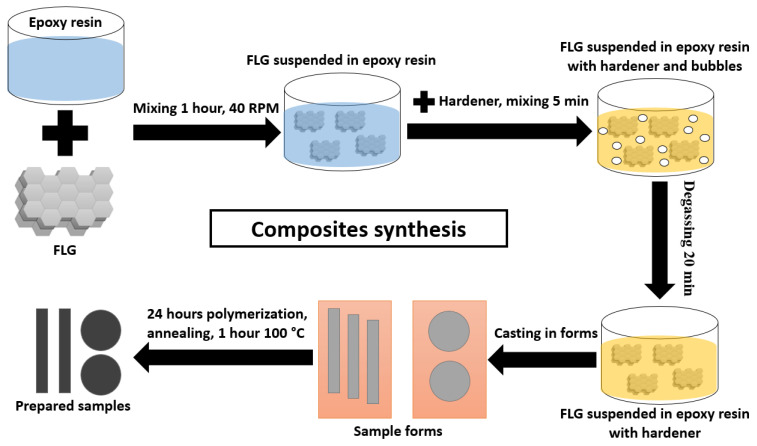
Scheme of synthesis of composites based on epoxy resin.

Composites based on epoxy resin were obtained according to the scheme in Figure 1. The initial epoxy resin was mixed with FLG using a top-drive agitator for an hour (40 RPM) at 45 °C. Then, the hardener triethylenetetramine was added and the mixture was mixed for 5 min. The mixture was then degassed in a vacuum chamber to remove air bubbles formed as a result of mixing. After degassing, the mixture was poured into molds and solidified for 24 h. The samples were then annealed in a muffle furnace for an hour at 100 °C to complete crosslinking of the epoxy resin. According to this method, samples were synthesized for testing flexural strength with a size of 80 mm × 12 mm × 2 mm, for tensile strength with a size of 150 mm × 10 mm × 3 mm, for compressive strength with a size of 6 mm × 5 mm × 3 mm, for the study of thermal conductivity with diameter d = 20 mm and thickness h = 100 mm, and for the study wear resistance diameter d = 40 and thickness h = 4 mm.

Images of FLG samples were obtained by transmission electron microscopy (TEM) using an FEI Tecnai G2 30 S-TWIN electron microscope (FEI Company, Hillsboro, OR, USA, 50 kV). X-ray analysis of FLG was performed on Rigaku smartLAB 3 X-ray diffractometer (Rigaku, Tokyo, Japan, CuK_α_ λ = 0.154051 nm). The IR spectra of FLG were obtained using an Infralume FT-08 spectrometer (Lumex Marketing, Saint Petersburg, Russia) by the method of attenuated total reflectance (ATR) on a PIKE attachment (Pike Technologies, Fitchburg, WI, USA). The Raman spectrum of FLG was studied using a Confotec NR500 spectrometer (NT-MDT, Moscow, Russia), which has an exciting laser wavelength of 532 nm. The particle sizes of FLG were determined by laser diffraction using Mastersizer 2000 (Malvern Panalytical, Malvern, UK). For measurements, a 50 mg sample was used, which was dispersed in 50 mL of deionized water by stirring for 1 min. Data on porosity and specific surface area were obtained by low-temperature nitrogen adsorption using Sorbi MS (META, Novosibirsk, Russia).

Optical images of the composites were obtained using a 1 mm sample layer mounted in a microscope Altami CMO870-T (Altami, Saint Petersburg, Russia) with the light source positioned underneath the sample. The samples’ flexural strength (ISO 178:2019 [28]), tensile strength (ISO 527-2:2012 [29]), and compressive strength (ISO 604:2002 [30]) were measured on a universal testing machine HSL-UT-50PC (Dongguan Hongjin Test Instrument Co., Dongguan, China) at a speed of 10 mm/min. The thermal conductivity at 25 °C was determined by the hot filament method on a Tempos (METER, Pullman, WA, USA). The composites’ glass transition temperatures were determined by the DSC method using BK-DSC300L (BIOBASE, Jinan, China), with a scanning speed of 5 °C/min.

**Figure 2:**
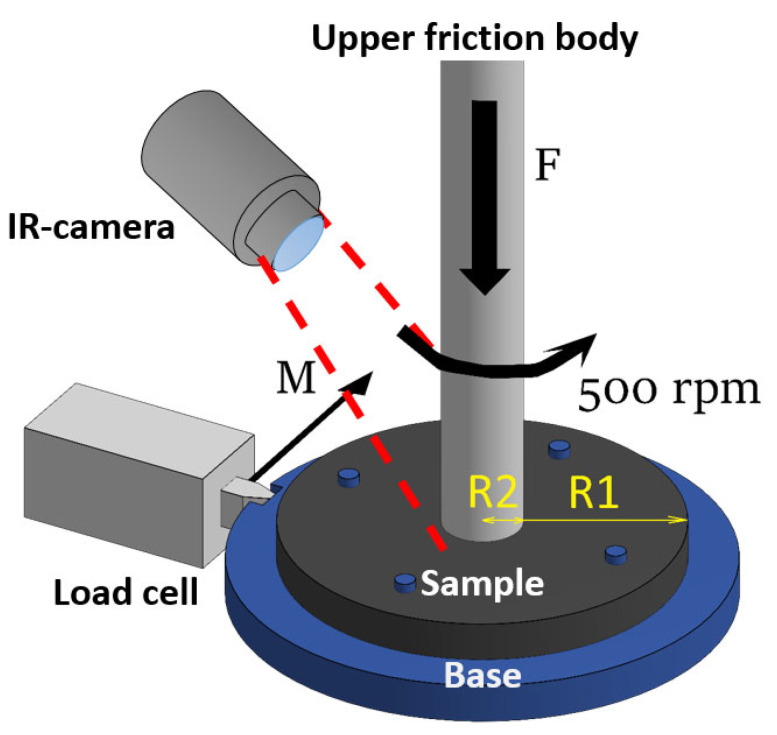
Scheme of the wear resistance testing, the red dotted lines show the camera’s viewing cone.

Wear resistance and coefficient of friction (steel/polymer) were determined on a universal friction machine UMT-200 (Converse-Resource, Moscow, Russia), disk-cylinder friction scheme (Figure 2). The upper body of rotation is pressed against the lower body (the test sample), which is rigidly mounted on the base. When the upper body rotates, the lower body and the base begin to rotate, which presses on the load cell from the data, of which the moment of force M is calculated. Further, the coefficient of friction was calculated using the equation:

µ = (M/F × R_1_/R_2_)

where M is the moment of force, F is the force of pressing the upper body against the lower one, R_1_ is the radius of the sample, and R_2_ is the radius of the friction imprint left by the upper body of rotation. Wear resistance was defined as the moment of destruction of the sample surface during friction with a sharp jump in the value of the coefficient of friction. An IR camera was also used to determine the temperature near the contact point of the friction pair. The pressing force of the upper body of rotation is 45 N, the diameter of the friction contact spot is 10 mm, and the rotation speed is 500 RPM.

## 3. Results

### 3.1. FLG from Biopolymers

**Figure 3:**
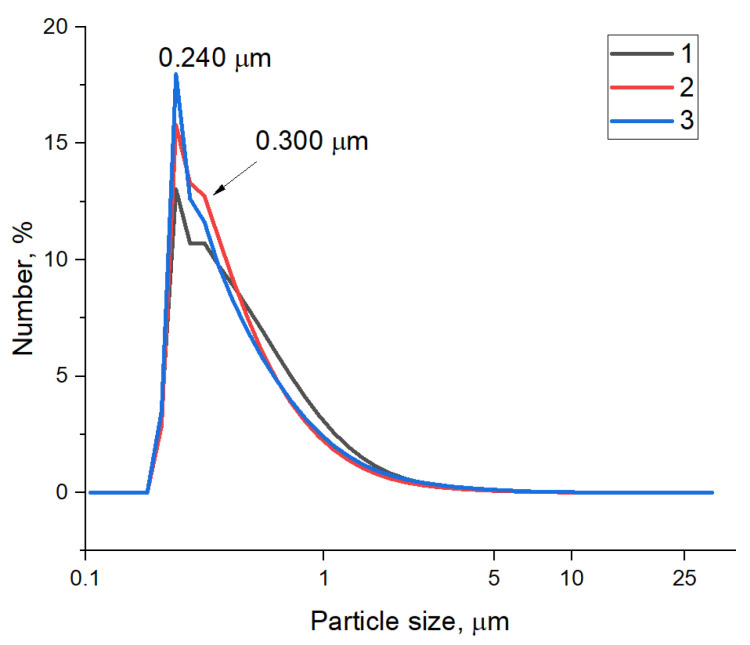
Particle size distribution of FLG samples obtained from cellulose (1), glucose (2), and lignin (3).

Synthesized samples of FLG were ground according to the procedure described in the experimental part, which allowed us to obtain a uniform distribution of FLG particles in the epoxy matrix. As a result of grinding, the average particle size for all samples ranged from 240 to 300 nm (Figure 3). It should be noted that a sample of FLG obtained from cellulose has a slightly higher proportion of large particles than the rest of the samples.

**Figure 4:**
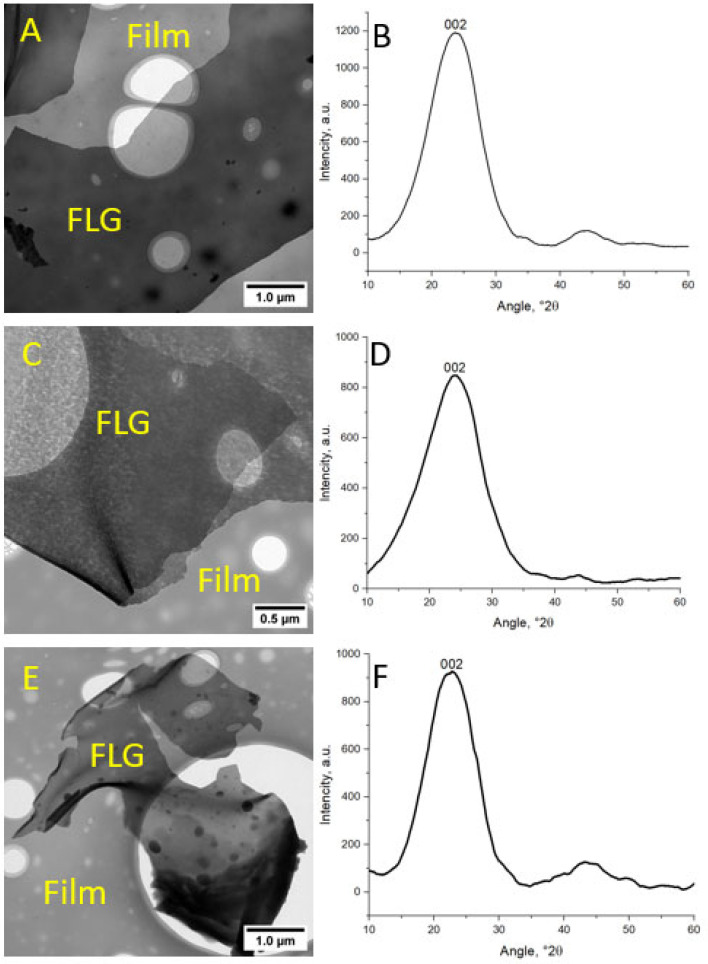
TEM images of FLG samples obtained from cellulose (**A**), glucose (**C**), and lignin (**E**); X-ray images of FLG samples obtained from cellulose (**B**), glucose (**D**), and lignin (**F**).

Figure 4A,C,E show TEM images of FLG samples obtained from cellulose, glucose, and lignin. It can be seen that the obtained samples are translucent particles. X-ray images of the obtained samples of FLG are shown in Figure 4B,D,F. Based on the peak width (002) at half-height, interplanar distances, and crystallite sizes were calculated: 3.74 Å and 9.28 Å for the cellulose sample, 3.7 Å and 7.89 Å for the glucose sample, and 3.94 Å and 9.4 Å for the lignin sample. Based on these data, according to the Scherrer equation, the number of layers in the samples does not exceed three. The absence of a peak at angle 2θ equal 10–11 in the diffraction patterns (Figure 4B,D,F) allowed us to exclude the formation of graphene oxide [31].

**Figure 5:**
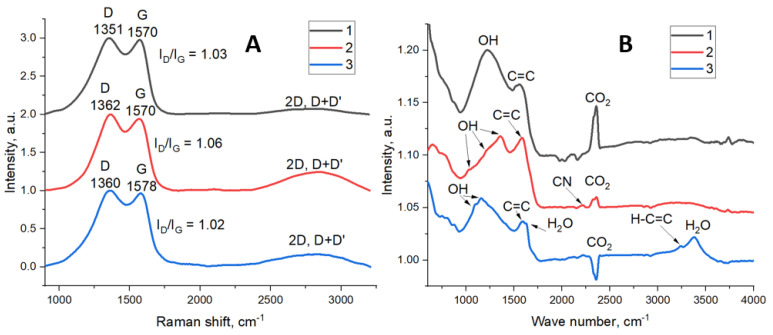
(**A**) Raman spectra of FLG from cellulose (1), glucose (2), and lignin (3); (**B**) FTIR spectra of FLG from cellulose (1), glucose (2) and lignin (3).

The Raman spectra of FLG samples (Figure 5A) are almost identical: the positions of the D and G bands, as well as their ratios of 1.03, 1.06, and 1.02 for FLG from the cellulose, glucose, and lignin, respectively. The Raman spectrum of the samples is similar to the spectra of graphene oxide and reduced graphene oxide [32]. This fact indicates a large number of defects in the structure of the samples—the presence of heteroatoms, sp3-carbon, and pores in the basal plane of graphene [33].

Figure 5B shows the FTIR spectra of cellulose, lignin, and glucose samples. A C=C coupling band is observed in all the spectra, and the wavenumber of this band depends on the sample and is equal to 1557 cm^−1^, 1583 cm^−1^, and 1590 cm^−1^ for FLG samples from cellulose, glucose, and lignin, respectively. The difference in the band position may be due to sp-3 carbon associated with aromatic cycles [34]. The FTIR spectra also show bands of the OH group: a wide band with a maximum of 1220 cm^−1^ for the sample from cellulose, three bands at 1351 cm^−1^, 1212 cm^−1^, and 1024 cm^−1^ for the sample from glucose, and bands at 1158 cm^−1^, and 1093 cm^−1^ for the sample from lignin. In addition, the sample from glucose has a low-intensity band of CN bond at 2220 cm^−1^. The presence of functional groups on the surface of the samples is due to the synthesis method, in which FLG is the product of the interaction of an organic substrate with ammonium nitrate.

The elemental composition of FLG samples was determined using the EDX method (Table 1). The presence of oxygen and nitrogen in the samples is due to the synthesis method, where the oxidizer NH_4_NO_3_ is used as an energy source. On the one hand, the samples have a similar surface composition, suggesting that self-propagating high-temperature synthesis results in a carbon material of a similar type (this is also evident from TEM and X-ray diffraction data). On the other hand, the samples differ in the composition of hydroxyl groups and the position of the C=C bond absorption band, which is probably due to the influence of the structure of the initial substrate and the synthesis of FLG itself. The FTIR method failed to detect functional groups containing nitrogen. However, the FLG sample from glucose exhibits a low-intensity band CN at 2220 cm^−1^. The bands responsible for the vibrations of nitrogen-containing groups can be hidden in wide bands responsible for the vibrations of hydroxyl groups.

The specific surface area and porosity of FLG are also essential factors in studying the effect of filler on the matrix in composite materials. These data are presented in Table 2. The values of the specific surface area of FLG powders obtained by self-propagating high-temperature synthesis significantly depend on the initial substrate. There are no sufficient grounds for discussing the dependence of the FLG powder structure on the specific surface area and porosity, and only the measured values of the specific surface area and pore volume will be considered.

### 3.2. Epoxy Composites

**Figure 6:**
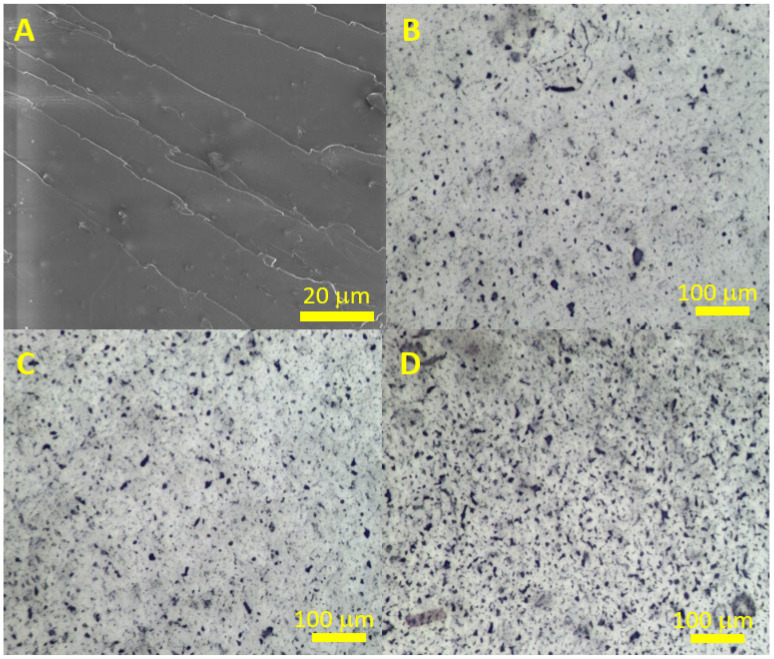
(**A**) SEM image of epoxy resin modified with FLG (1 wt.%); Optical images of epoxy resin modified with FLG: (**B**) 0.25 wt.%, (**C**) 0.5 wt.%, (**D**) 1 wt.%.

To study the distribution of FLG in the epoxy resin, we made an attempt to obtain SEM images of the composites. Figure 6A shows the SEM image of epoxy resin containing 1 wt.% FLG. From this image, it is not possible to determine the nature of the distribution of FLG particles in the volume of the matrix. Therefore, images were obtained from an optical microscope (Figure 6B–D) where the light source was located under the sample, shining through the sample. From the optical images, it is evident that FLG particles exist in the epoxy resin both as individual particles (gray areas) and as clusters (black areas). With increasing concentration, the proportion of black areas in the image increases, which is associated with the aggregation of FLG particles.

**Figure 7:**
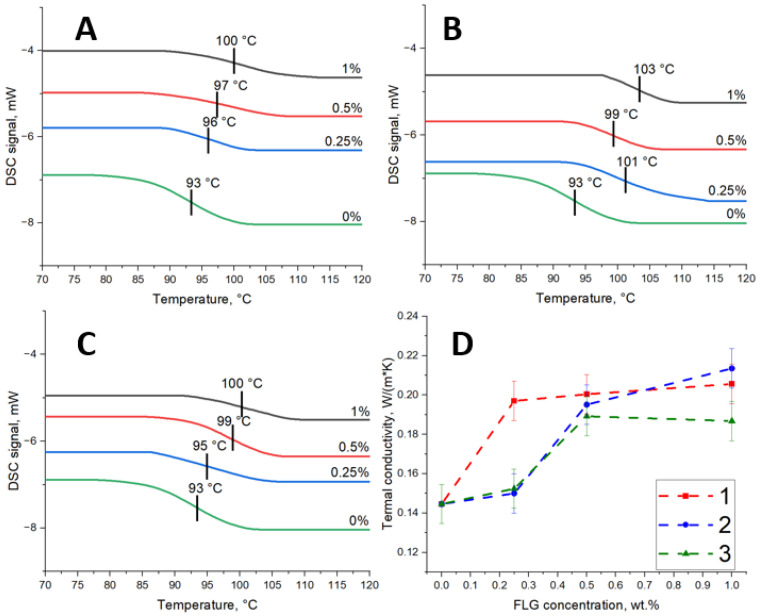
DSC curves of composites based on epoxy resin modified with FLG obtained from cellulose (**A**), glucose (**B**), and lignin (**C**); (**D**) Dependence of the thermal conductivity of composites based on epoxy resin on the concentration of FLG obtained from cellulose (1), glucose (2) and lignin (3).

Using the DSC method, it was found that adding FLG increases the epoxy resin’s glass transition temperature (Figure 7A–C). When the concentration of FLG is increased to 1 wt.%, the glass transition temperature increases from 93 °C to 100–103 °C, depending on the FLG sample’s origin. When the glass transition temperature is exceeded, individual segments of the polymer chain become mobile, and the polymer goes from a glassy state to a highly elastic state. An increase in the glass transition temperature with the addition of FLG indicates that FLG obstructs the movement of the polymer chain and needs to give more energy to the polymer to overcome this resistance. Due to functional groups, the FLG probably interacts with the hydroxyl groups of the epoxy resin and the amine groups of the hardener. A similar result was observed in the work devoted to modifying nitrile butadiene rubber by GNS [35].

Figure 7D shows that the thermal conductivity of composites based on epoxy resin modified with FLG is higher than that of pure epoxy resin. When the concentration of FLG is 1 wt.%, the average thermal conductivity of the samples increases to 40%. Considering the good adhesion of FLG to the matrix due to functional groups at 1 wt.%, one would expect a more significant increase in the thermal conductivity of the composite. For example, in [36], authors also provided a good interaction of reduced graphene oxide obtained by the Hammers method with epoxy resin, which made it possible to achieve an increase in thermal conductivity by 80% (25 °C) at 1 wt.%. However, neither in [36] nor in other similar works [37,38] experimentally measured thermal conductivity of composite materials does not correspond to that calculated according to theoretical models, which is usually associated with particle aggregation. Also, we should not forget that when the number of defects in the graphene structure increases and the particle size decreases, its thermal conductivity decreases [39]. Considering the particle size and many defects (according to Raman spectra), the thermal conductivity increases by 40% at 1 wt.% and can be regarded as satisfactory when using FLG obtained by self-propagating high-temperature synthesis.

**Figure 8:**
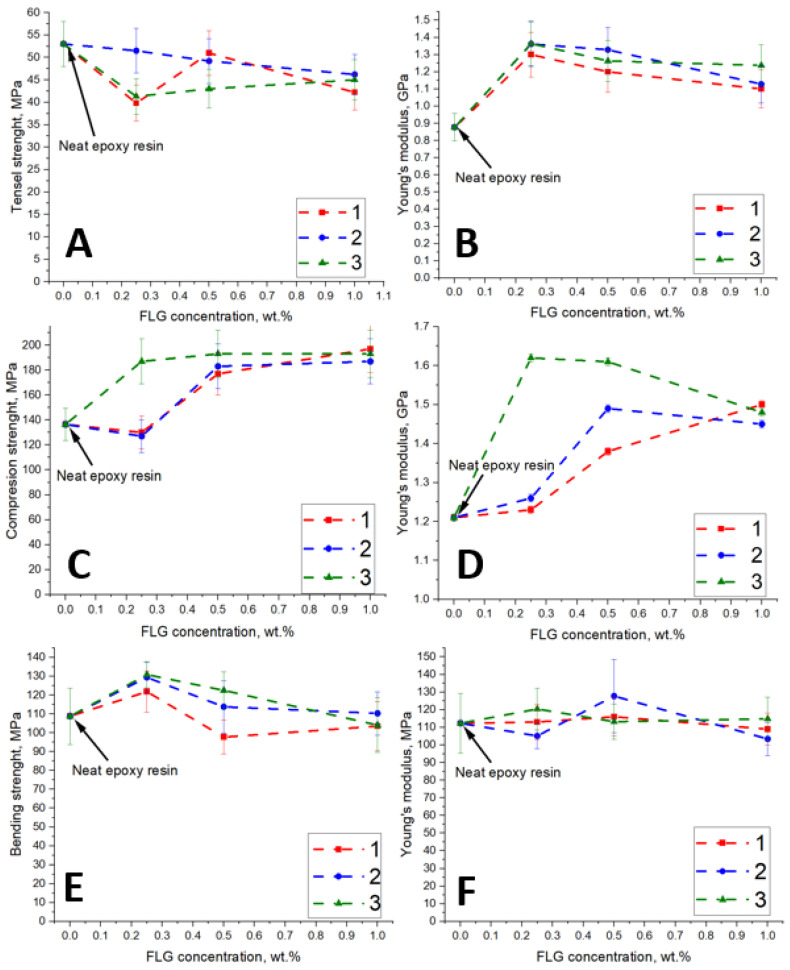
Dependence of the tensile strength (**A**), compressive strength (**C**), bending strength (**E**), and the elastic modulus of the tensile strength (**B**), compressive strength (**D**), and bending strength (**F**) of epoxy resin on the concentration of FLG obtained from cellulose (1), glucose (2) and lignin (3).

The strength data of composites based on epoxy resin modified with FLG are shown in Figure 8. It can be seen that the tensile, flexural, and compressive strengths have the same dependences relative to the origin of FLG. An increase in the concentration of FLG leads to a decrease in the tensile strength of the epoxy resin (Figure 8A) and an increase in the Young’s modulus (Figure 8B). At a concentration of 1 wt.%, the tensile strength of epoxy resin is reduced by 15%, while the elastic modulus increases by 30%. Thus, an epoxy resin-modified FLG breaks down more brittle than pure epoxy resin, which was also noted in other works [40,41]. At the interface between the matrix and FLG, epoxy resin molecules can be structured to some degree to the total volume of the polymer due to adhesion. As a result of this process, stresses arise, and breaks can occur in these areas when stretched.

FLG increases the compressive strength of epoxy resin. At an FLG concentration of 1 wt.%, the compressive strength increases by 40% (Figure 8C) and the Young’s modulus by 20%. (Figure 8D). When compressed, the distance between the molecules decreases, and the polymer chains begin to move perpendicular to the pressure axis. In the movement of molecules, particles of FLG act as obstacles and areas of concentration for polymer molecules, resulting in the self-strengthening of the material. It can be noted that the compressive strength of the epoxy resin modified with FLG from lignin increases already at a concentration of 0.25 wt.%, while the Young’s modulus of the composite also increases by 30%. This difference may be due to the initial substrate used for the synthesis of FLG. It can be assumed that FLG obtained from cellulose and glucose (saccharides) differs in structure from FLG from lignin (polyphenol), but this issue requires further research.

The bending strength of the epoxy resin increases by 20% (Figure 8E) at a FLG concentration of 0.25 wt.%. With a further rise in FLG concentration, the composite strength decreases to the initial values of pure epoxy resin. When bending, the upper layers of the sample are compressed, and the lower layers are stretched. Thus, with the introduction of FLG, the decreasing tensile strength is offset by the increasing compressive strength, which practically does not change the bending strength.

Composites based on epoxy resin modified with rGO were also obtained. The strength and thermal conductivity data were compared with those of the composite modified with FLG from lignin (Table 3). The use of rGO as a filler in epoxy resin results in a slight increase in the tensile strength of the epoxy resin. However, rGO does not have a significant effect on the compressive and flexural strengths, as well as thermal conductivity. It can be assumed that rGO obtained by reducing GO has very few functional groups to ensure good adhesion to the epoxy matrix.

**Figure 9:**
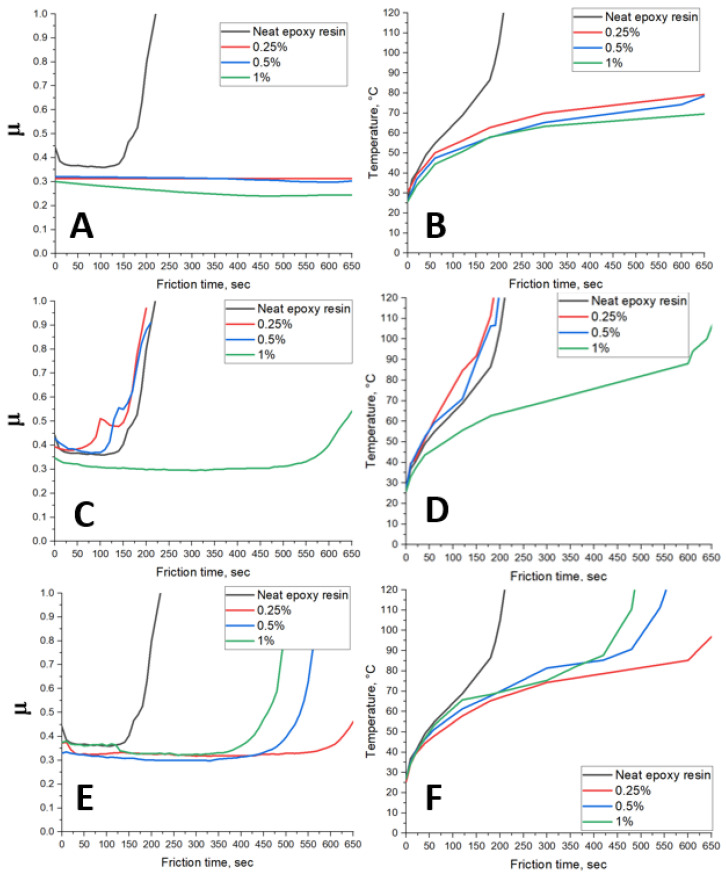
Dependence of the friction coefficient of the steel/epoxy resin pair on the friction time at a concentration of FLG of 0, 0.25, 0.5, 1 wt.% obtained from cellulose (**A**), glucose (**C**), and lignin (**E**); Dependence of the temperature in the vicinity of the friction contact spot on the friction time at a concentration of FLG of 0, 0.25, 0.5, 1 wt.% obtained from cellulose (**B**), glucose (**D**), and lignin (**F**).

FLG can reduce the friction coefficient of the steel/epoxy pair and increase its wear resistance. Figure 9 shows the dependences of the coefficient of friction (μ) on the test time of composites based on epoxy resin modified with FLG obtained from cellulose (Figure 9A), glucose (Figure 9C), and lignin (Figure 9E). The dependence of the temperature in the vicinity of the friction spots on the friction time of composites is based on epoxy resin modified with FLG obtained from cellulose (Figure 9B), glucose (Figure 9D), and lignin (Figure 9F). Introducing FLG obtained from cellulose into the epoxy resin leads to a decrease in the friction coefficient of the steel/epoxy resin pair from 0.37 to 0.31 at concentrations of 0.25 and 0.5 wt.%. Increasing the concentration to 1 wt.% reduces the coefficient of friction to 0.25. At the same time, FLG obtained from cellulose increases the wear resistance of epoxy resin. The neat epoxy resin breaks in 150 s after the start of the experiment, and the epoxy resin modified with FLG obtained from cellulose did not break in 600 s. The graph of the temperature dependence of composites modified with FLG from cellulose (Figure 9B) shows that with an increase in the concentration of FLG obtained from cellulose, the temperature during friction increases more slowly. At 600 s of testing, the temperature near the friction spot for composites is 78 °C, 74 °C, and 68 °C for concentrations of 0.25 wt.%, 0.5 wt.% and 1 wt.%, respectively. The decrease in the coefficient of friction can be attributed to the fact that FLG fills in the surface roughness during friction [42]. The equation can explain slower heating during friction:

*Q* = *µ* × *F* × *V* × *t*,

where *Q* is heat generated during friction (J), *µ* is the coefficient of friction, *F* is the force of pressing the upper body against the lower one (N), *V* is the speed (m/s), *t* is time (s). Thus, a decrease in the coefficient of friction led to a reduction in heat generation during friction and, consequently, a decrease in the heating rate.

The effect of reducing the friction coefficient of the steel/epoxy resin pair when using FLG obtained from glucose is observed only in a concentration equal to 1 wt.% (Figure 9C). The coefficient of friction at 1 wt.% of FLG obtained from glucose decreases from 0.37 to 0.3, and the time to destruction of the sample increases from 150 s to 600 s. Here, the percolation effect is observed, in which up to a certain concentration, in our case below 1 wt.%, there is no effect on the tribological properties of the epoxy resin. Judging by the structural data presented at the beginning of the discussion, the values of the specific surface area and pore volume can be an influencing parameter in this process. The powder of FLG obtained from glucose has the smallest specific surface area and pore volume compared with samples obtained from lignin and cellulose. It is necessary to consider the total value of the surface of FLG (which must be bonded with epoxy resin) to reduce the coefficient of friction.

The opposite effect is observed when using FLG obtained from lignin (Figure 9E). When it is introduced into an epoxy resin with a concentration equal to 0.25 wt.%, there is a decrease in the coefficient of friction from 0.37 to 0.32 and an increase in wear resistance from 150 to 600 s. Increasing the concentration of FLG obtained from lignin from 0.25 wt.% up to 1 wt.% wear resistance decreases from 600 s to 375 s, which is also reflected in the heating curves (Figure 9F). At the same time, the coefficient of friction remains almost at the same level. In this case, it is impossible to determine the factor that affects the decrease in wear resistance of the composite with an increase in the concentration of FLG obtained from lignin. Wear resistance can be considered a process of surface fatigue, in which, due to friction, microcracks accumulate on the sample surface. When a specific concentration of microcracks is reached, the material is destroyed [43]. FLG reduces the friction coefficient of the steel/epoxy pair, which reduces the mechanical impact on the surface of the epoxy resin.

At low concentrations of FLG in epoxy resin (0.25 wt.%), we observed a correlation between the specific surface area of FLG and the wear resistance of the composite. FLG obtained from glucose has the smallest measured specific surface area and the wear resistance of the composite with this FLG sample is the same as that of neat epoxy resin. The FLG sample obtained from cellulose has the largest specific surface area, and the epoxy resin modified with FLG from cellulose did not brake during the experiment—it has the greatest wear resistance. FLG from lignin has an intermediate specific surface area as well as the composite modified with this sample. Thus, it can be stated that the higher the value of the specific surface area of the FLG powder, the more wear-resistant the composite becomes, probably due to the greater coverage of the friction surface FLG.

At high concentrations of FLG in epoxy resin (1 wt.%), the dependence of composite wear resistance on specific surface area becomes unclear. For composites modified with FLG from cellulose and glucose, wear resistance remains predictable. By increasing the concentration of FLG obtained from cellulose in epoxy resin, the friction coefficient and heat released as a result of friction are reduced. This occurs due to the increase in FLG concentration with a large specific surface area, as a result the area of FLG participating in the friction process increases significantly. Increasing the concentration of FLG obtained from glucose also increases the wear resistance of the epoxy resin upon reaching certain value of FLG involved in the friction process. Increasing the concentration of FLG obtained from lignin reduces the wear resistance of the composite. This phenomenon cannot be associated with any structural features of the FLG obtained from lignin. It may possibly be associated with the processes of secondary aggregation of few-layer graphene particles as a result of friction.

## 4. Conclusions

It was found that few-layer graphene produced by self-propagating high-temperature synthesis significantly enhances the overall mechanical (compressive strength, flexural strength), thermophysical (thermal conductivity), and tribotechnical (wear resistance) properties of the final epoxy-based composite. During experimental comparison, few-layer graphene demonstrated greater effectiveness than reduced graphene oxide.

It was also established that the effectiveness of few-layer graphene in epoxy composites depends on the type of precursor used for its synthesis. This finding is an unexpected result and will be further investigated in subsequent studies.

## Figures and Tables

**Table 1:** Elemental composition of FLG samples.

Sample	C, at. % ± 1%	O, at. % ± 1%	N, at. % ± 1%
FLG from cellulose	84	6	10
FLG from glucose	78	11	11
FLG from lignin	89	7	4

**Table 2:** Specific surface area and pore volume of FLG samples.

Sample	BET Sample, m^2^/g ± 6%	Vpore, mL/g ± 6%	Vmicro, mL/g ± 6%
FLG from cellulose	340	0.225	0.12
FLG from glucose	75	0.109	0
FLG from lignin	238	0.188	0.05

**Table 3:** The greatest increase in strength and thermal conductivity obtained in epoxy resin using FLG from lignin and rGO.

Characteristic	FLG from Lignin	rGO
Tensile strength	−15% (0.5 wt.%)	+10% (0.5 wt.%)
Compressive strength	+40% (0.5 wt.%)	+15% (0.5 wt.%)
Bending strength	+15% (0.5 wt.%)	+7% (0.5 wt.%)
Thermal conductivity	+40% (0.5 wt.%)	+12% (0.5 wt.%)

## Data Availability

The data presented in this study are available on request from the corresponding author.

## Abbreviations

The following abbreviations are used in this manuscript:

FLG	Few layers graphene
GNS	Graphene nanostructure
GNP	Graphene nanoplates
GO	Graphene oxide
rGO	Reduced graphene oxide
